# Preoperative patient teaching practices and associated factors among nurses working at hospitals in West Shoa Zone, Ethiopia, 2022: a cross-sectional study

**DOI:** 10.3389/fpubh.2024.1498406

**Published:** 2024-12-18

**Authors:** Lammi Atomsa, Sidise Temesgen, Abebe Dechasa, Mulatu Ayana, Nimona Amena, Dawit Teklehymanot, Firaol Regea

**Affiliations:** ^1^Department of Nursing, School of Nursing and Midwifery, Wallaga University, Nekemte, Ethiopia; ^2^College of Health Sciences, Ambo University, Ambo, Ethiopia; ^3^Department of Nursing, College of Health Sciences, Medawelabu University, Robe, Ethiopia

**Keywords:** preoperative teaching, practice, nurses, patient, Ethiopia

## Abstract

**Introduction:**

Preoperative teaching is fundamental nursing activity in which a nurse educates the patient about surgery and what to anticipate following the procedure. It is a process via which nurses give standard preoperative information to patients before surgery, and it enables the patients to understand their diagnosis and treatment, actively participate in their own care, overcome feelings of incapacity in relation to their condition, regain health, and maintain home care. However, there is a dearth of studies that determine the extent of preoperative teaching practice in Ethiopia in general and in the study area in particular.

**Objective:**

This study aimed to assess preoperative patient teaching practices and the factors associated with these practices among nurses working in hospitals in the West Shoa, Oromia region, Ethiopia, in 2022.

**Methods:**

An institution-based cross-sectional study was conducted among 267 nurses from 1 September to 30 September 2022, at hospitals in the West Shoa Zone. Two-stage simple random sampling technique was used to select study participants. Data were collected using a pretested and structured self-administered questionnaire. The quantitative data were checked and entered using Epi-data version 4.6 and exported to SPSS version 26.0 for analysis. Bivariable and multivariable logistic analyses were performed, and *p*-values of <0.05 at a 95% confidence interval were considered statistically significant.

**Result:**

A total of 253 nurses returned the entire questionnaire, yielding a response rate of 94.75%. The study enrolled 132 (52.2%) male, the highest percentage (231, 91.3%) of the participants were in the age group of 18–35, the majority of participants (152, 60.1%) were married, and 164 (64.8%) were protestant. Approximately 101 (39.9%) participants demonstrated good preoperative teaching practice. Lack of teaching material, lack of training workload, time constraints, insufficient staffing, language barrier, severity of patient cases, patient and family’s anxiety, and complexity of patients’ status were significantly associated with preoperative patient teaching practice.

**Conclusion:**

The proportion of preoperative patient teaching practices among nurses working at hospitals in the West Shoa Zone was low. Concerned bodies should emphasize ways to improve preoperative teaching practice.

## Introduction

The preoperative period is the time between the decision to have surgery and the patient’s transport to the operating room (1). The preoperative period is one of the most stressful times, resulting in mild-to-severe anxiety (2, 3). Patient teaching, as part of professional nurses’ educational responsibilities, ensures that healthy knowledge, attitudes, behaviors, and habits are learned by both healthy and sick individuals. Today’s patient teaching has lately moved from a concentration on sickness pathophysiology and treatment to one that encompasses disease prevention, patient development, and patients family care, as well as their engagement in care management (4).

Patients awaiting anesthesia and surgery are more likely to experience worry and fear (5). The prevalence of preoperative anxiety, reported in previous studies, was 60–80% of which 59.6% of them are afraid that they will not recover from anesthesia; 53.9% are afraid of mortality, dependency, and impairment; 51.7% are afraid of postoperative pain; and 43.3% are worried about their families (6, 7).

Preoperative anxiety is a psychological condition that frequently precedes surgery and may have a negative impact on the postoperative outcome (8). Patient with preoperative anxiety might have operation-related complication such as increased autonomic nerve fluctuations, a greater need for anesthesia, a higher risk of nausea and vomiting, and increased postoperative pain. Reports state that these issues may extend the length of the hospital stay and the rehabilitation period (8, 9).

Preoperative teaching is a process via which nurses give standard preoperative information to patients before surgery (10). Preoperative teaching enables the patients to understand their diagnosis and treatment, actively participate in their own care, overcome feelings of incapacity in relation to their condition, regain health, and maintain home care (11).

The main topics about which patients and their relatives should be informed before an operation are preoperative diagnosis, preparation, treatment, duration of operation, material to be used, frequency and duration of relatives’ visits, location of relatives’ waiting room, method of communication and how to share information with staff on the postoperative ward, and tubes to be inserted to the patient (12).

Preoperative teaching is crucial for alleviating fear and anxiety about surgery, relieving patient concerns, and preventing postoperative problems (13). It is also important to help clients better comprehend their surgical treatment, feel more in control, experience less postoperative discomfort and anxiety, spend less time in the hospital, and recover quickly (14). In addition, effective preoperative teaching could save approximately 5.7 million US$ cost of postoperative period per year (15).

Failure to provide adequate preoperative information causes anxiety, fear of pain, and uncertainty about the future, depression, anger, and inability to perform personal functions after the operation. As a result, both the risk of complications and length of hospital stay increase (3, 16). In this regard, all healthcare providers, particularly nurses, should recognize that the patient requires information on the procedures that occur before, during, and after the operation and incorporate preoperative education into patient care (17).

Recent studies reported that the level of preoperative teaching varies on different continents of the world and that implementation is low in developing countries (18–21). Some studies revealed that most of the nurses have a good knowledge of preoperative patient teaching and a positive attitude toward it, and those studies found a significant gap between knowledge and practice among the study participants. According to these studies, although the nurses have good knowledge, their practice is poor (22).

Considering preoperative teaching as a major duty of nurse professionals, investigating the extent of its practice found it to be one factor that has received little attention in various countries, including Ethiopia (23). To the best of our knowledge, few studies have assessed the practices and associated factors of preoperative patient teaching in Ethiopia. Thus, the researchers intended to assess preoperative patient teaching practice and associated factors among nurses working at hospitals in the West Shoa Zone, Ethiopia.

### Conceptual framework

**Figure 1:**
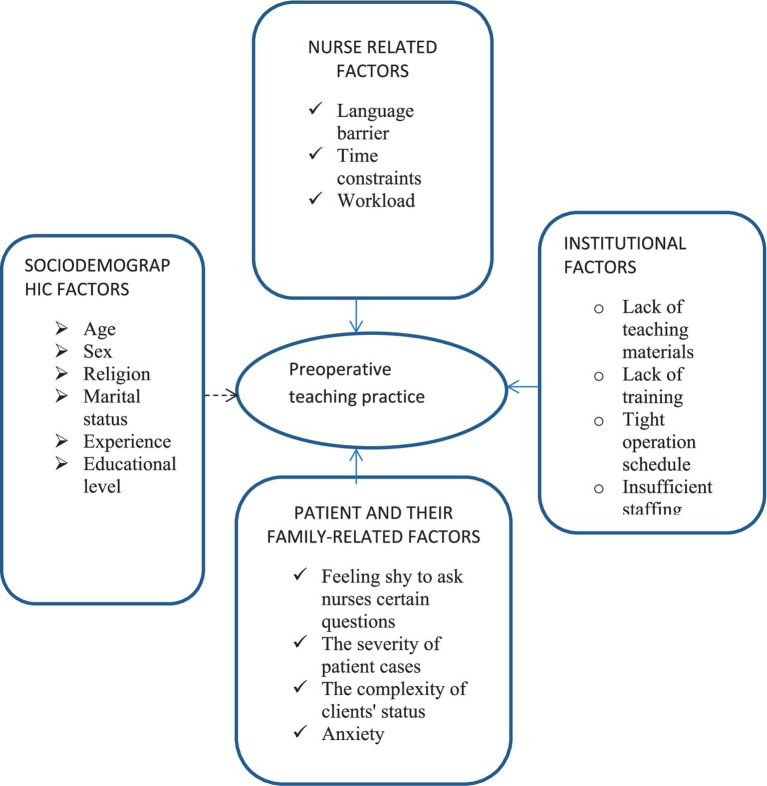
Developed conceptual framework for preoperative teaching practice and associated factors among nurses working at hospitals in West Shoa Zone, Ethiopia, 2022.

The conceptual framework was developed based on the review of different studies. Factors such as sociodemographic factors, nurse-related factors, institutional-related factors, and patient- and family-related factors are those determinant factors which can affect the preoperative teaching practice either proximally, intermediately, or distally (12, 14, 24–27) (see Figure 1).

### Objectives

The general objective of the study was to assess preoperative patient teaching practices and the factors associated with these practices among nurses working in hospitals in the West Shoa, Oromia region, Ethiopia, in 2022. The specific objectives the study are to determine the level of preoperative patient teaching practice and identify factors associated with preoperative patient teaching practices among nurses working at hospitals in the West Shoa Zone, Oromia region, Ethiopia, respectively.

## Methods and materials

### Study area and period

The study was conducted from 1 September to 30 September 2022, at hospitals in the West Shoa Zone, Oromia, Ethiopia, 2022. The zone now has nine hospitals, including primary, secondary, and tertiary hospitals. All hospitals provide surgical services to patients.

### Patient and public involvement

The research questions and study design were created by the investigators, who then got them approved by the Ambo University’s institutional review board. None of the participants in this study were involved in its design, implementation, or dissemination techniques.

### Study design

An institution-based quantitative cross-sectional study was employed.

### Population

The study’s source population consists of all nurses working in hospitals located in the West Shoa Zone. The study’s population consists of all sampled nurses who work in selected hospitals in the study area and coincide with the inclusion criteria. Individual nurses working at chosen hospitals in the study area were the study’s unit.

#### Inclusion criteria and exclusion criteria

Nurses available during the data collection period who volunteered to participate were included in the study. Specialty nurses (such as psychiatry nurses, ophthalmologic nurses, and emergence and critical care nurses) were excluded because they were not assigned to the surgical ward or operating room during nursing rotation was excluded from the study.

### Sample size determination and sampling procedure

#### Sample size determination

Sample size was determined using a single population proportion formula by the following assumptions: Z = the standard normal deviation at a 95% confidence interval = 1.96 and d = margin of error that can be tolerated, 5% (0.05). The sample size was calculated by assuming p 0.5(50%) since there are no similar studies being conducted in Ethiopia.


$$n=\frac{{\left(Z\alpha /2\right)}^{2}*P\left(1-P\right)}{d^{2}}n=\frac{{\left(1.96\right)}^{2}*0.5\left(1-0.5\right)}{\left(0.05\right)2}=384$$

However, because this is population of <10,000, the corrected sample size was calculated using the following formula:


$$n_{f}=\frac{n}{1+n/N}=\frac{384}{1=384/657}=243$$

where

- *N* = total number of nurses at hospitals in West Shoa Zone =657
- *n* = desirable sample size required for the study
- Z (*α*/2) = the critical value at 95% level of significance (1.96)
- P = proportion of nurses who demonstrate good preoperative teaching practices
- d = margin of error (5%).

Assuming 10% non-response rate, the final sample size was 267.

#### Sampling procedure and technique

From a total of nine hospitals, four were selected to participate (Ambo University Referral Hospital, Gedo General Hospital, Incini Primary Hospital, and Gudar Primary Hospital) by simple random sampling technique. Next, sample size was proportionally allocated to the selected hospitals based on the number of nurses. Finally, simple random sampling method was used to select proportionally allocated study participants from each hospital using the lottery method.

Proportional allocation was calculated using the following formula:


ni=n/N

where

**Figure 2:**
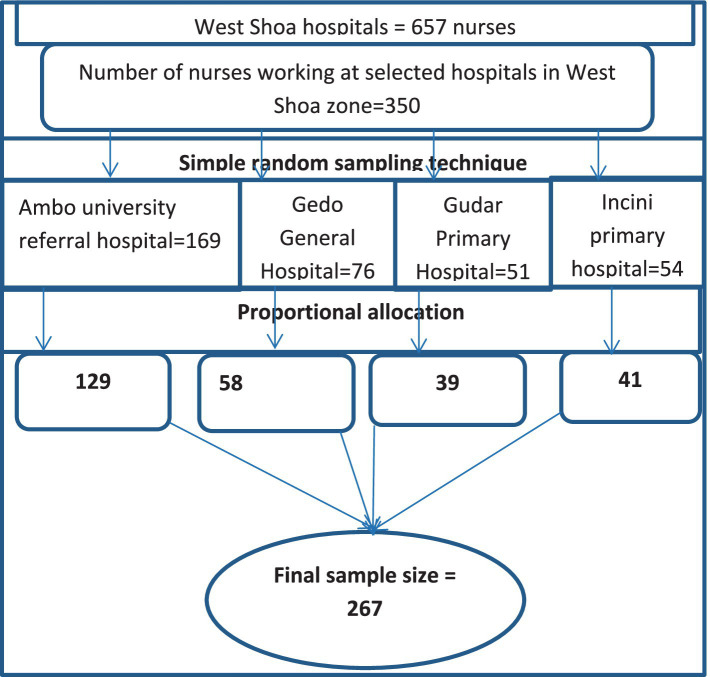
Schematic representation of sampling technique for preoperative patient teaching practice and associated factors among nurses working at hospitals in West Shoa Zone, Ethiopia, 2022.

- ni = the sample size of i th hospital
- n = n1 + n2 + …n4 is the total sample size
- Ni = the population size of i th hospital
- N = N1 + N2 + N3 + N4 is total hospital population (see Figure 2).

Simple random sampling technique was used to select study participants.

### Variables

Independent variable of study was preoperative teaching practice.

Independent variables of study were as follows:

Sociodemographic factors: age, sex, marital status, religion, work experience, and educational level.

Institutional factors: lack of teaching materials, lack of training, tight operation schedule, and insufficient staffing.

Nurse-related factors: workload, language barrier, and time constraints.

Patient- and family-related factors: feeling too shy to ask nurses certain questions, severity of patient cases, complexity of clients’ health status, and anxiety.

### Operational definitions

Good preoperative teaching practice: The participants answered “always” to all practice questions (14, 28, 29).

Poor preoperative teaching practice: Participants did not reply “always” to all practice questions.

### Data collection tools and procedures

A structured self-administered questionnaire was used to collect the necessary data from study participants. The questionnaire was adapted after reviewing different articles (10, 12, 14, 25, 30–32) and comprised three sections: The first section concerned the sociodemographic characteristics of the participants, and the second part consists of a Likert scale designed to measure nurses preoperative teaching practice. The third section consisted of a Likert scale designed to measure perceived barrier to preoperative education. The two-point Likert scale is the simplest Likert scale question, for example, where there will be just two Likert options, such as agree and disagree as two poles of the scale. It is typically used to measure agreement. Because we intended to assess their agreement on such barriers, we used two-point Likert scales. The scale consisted of 11 questions to which participants responded either (Agree = 1 or Disagree = 2). One supervisor and four data collectors with BSc degree in nursing were recruited for data collection process. Prior to administering the questionnaire, the data collectors informed the study participants about the purpose of the study data collectors devoted as much time as possible to encouraging participants to respond to all items in the questionnaire.

### Data quality assurance and control

One week before actual data collection, the questionnaire was pretested on 5% of the total sampled population working at Holeta Hospital to check clarity and consistency and make necessary amendments. Any ambiguity, confusion, difficult words, or differences in understanding were revised based on the pretest experience. The consistency and reliability of this survey were measured by Cronbach’s alpha which was 0.94 and 0.803 for the practice and barriers questions, respectively. Before the actual data collection period, supervisors and data collectors were oriented on the objective of the study, the questionnaire and extent of explanations, and the way to maintain privacy and confidentiality. The supervisor and investigators coordinated the data collection process.

To ensure data quality, training was provided to the data collectors and supervisor on the study objectives and questionnaires. The data collectors were supervised by the supervisor, and the investigator received a daily report. All data were checked for completeness and consistency by the investigator and supervisors on the day of data collection. Double data entry was performed to ensure the quality of data; data were intensively cleaned before analysis.

### Data processing and analysis

The data were coded and entered using Epi-data version 4.6 and exported to SPSS version 26.0. Categorical variables are summarized as frequencies and percentages. To measure the association between the outcome and independent variables, adjusted odds ratios (AORs) with 95% confidence interval were calculated. To identify factors associated with the outcome variable, a bivariate logistic regression analysis was first performed for one independent variable with the outcome variable. Variables with a *p*-value of less than 0.25 were considered potential candidates for multivariable logistic analysis. The multi-collinearity test was performed with a variance inflation factor (VIF), with maximum of 1.2. Model goodness of fit was tested using Hosmer–Lemeshow goodness-of-fit test. A forward stepwise (likelihood ratio) method was used. Statistical significance was declared for variables with a *p*-value of less than 0.05.

### Ethical considerations

Ethical clearance was obtained from the ethical review board (ERB) of Ambo University, College of Medicine and Health Sciences. Subsequently, a supporting letter was sent to all hospitals to conduct this research in the hospitals that were involved in this research, and written consent was obtained from respondents before data collection. All study participants were informed about the purpose of the study and their right to participate and were assured that their names would not be visible on their responses to the questionnaire to maintain their confidentiality.

## Results

### Sociodemographic characteristics of participants

A total of 253 nurses were returned completed questionnaire making a response rate of 94.75%. The study enrolled 132 (52.2%) male, most participants (231, 91.3%) were in the18–35 age group, the majority (152, 60.1%) were married, and 164 (64.8%) were protestant. The majority (210, 83.0%) of respondents had a Bachelor of Science in nursing, and 181 (78.5%) had worked in the nursing profession for 9 years or less (Table 1).

**Table 1:** Sociodemographic characteristics of selected nurses working at hospitals in West Shoa, Ethiopia, 2022.

Variables	Frequency	Percent
Age groups		
18–35	231	91.3
≥35	22	8.7
Total	253	100.0
Sex		
Male	132	52.2
Female	121	47.8
Total	253	100.0
Marital status		
Single	88	34.8
Married	152	60.1
Others	13	5.1
Total	253	100.0
Religion		
Orthodox	73	28.9
Protestant	164	64.8
other	16	6.3
Total	253	100.0
Level of education		
Diploma in Nursing	23	9.1
BSc in Nursing	210	83.0
MSc in Nursing	20	7.9
Total	253	100.0
Work experience		
≤9 years	181	71.5
≥10 years	72	28.5
Total	253	100.0

### Distribution of respondents’ preoperative patient teaching practice

Among 253 participants, the majority (162, 64%) always taught the patients about routine surgical procedures before surgery; 52 (20.2%) rarely taught patient’s family members before surgery; 153 (60.5%) taught patients within specific time period before surgery; and 159 (62.8%) always taught patients about preoperative fasting before undergoing surgery. Approximately 50.6% of participants always use different methods when they provide preoperative teaching to patients before surgery. Approximately 166 (65.6%) of participants always informed the patients about the postoperative dietary requirements. Approximately 153 (60.5%) of survey participants always explained the forms of anesthetic to be used before surgery. 157 (62.0%) individuals offer instruction on bladder emptying before going to the theater.

**Figure 3:**
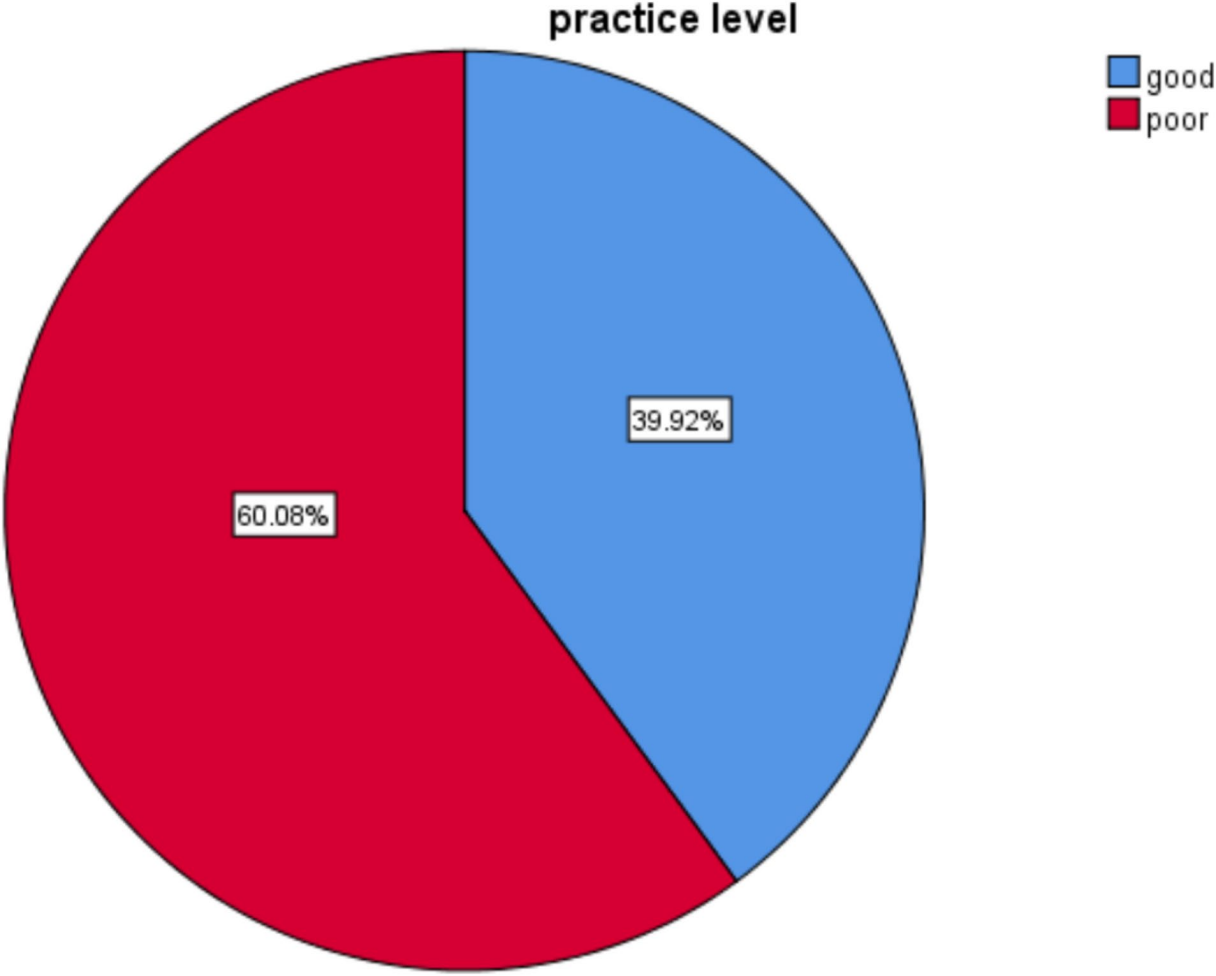
Level of practice on preoperative patient teaching among nurses working at hospitals in West Shoa Zone, Ethiopia, 2022.

Of participants, 101 (39.9%; 95% CI: 33, 46) responded ‘Always’ to all 19 questions, demonstrating that they followed good practice. The remaining (132, 60.1% with 95% CI: 54, 66) participants demonstrate that they followed poor practice (Figure 3).

### Participants perceived barriers to preoperative education practice

Among 253 participants, almost 148 (58.5%) of study participants agreed that the complexity of patients’ status influences the practice of preoperative patient teaching practice. The majority (142, 56.1%) agreed that lack of teaching materials used for preoperative teaching could affect the delivery of preoperative patient teaching. More than half (133, 52.6%) agreed that lack of training is a factor affecting delivery of preoperative education to patients before undergoing surgery, 131 (51.8%) agreed that it was affected by the daily workload of the nurse, and approximately 140 (55.3%) agreed that lack of time among nursing staff could also affect delivery. The majority (142, 56.1%) agreed that shortage of nursing staff was a factor affecting the practice of preoperative patient teaching. Almost all (148, 58.5%) of study participants agreed that the complexity of patients’ status influences the practice of preoperative patient teaching practice (Table 2).

**Table 2:** Perceived barriers of preoperative patient teaching practice among nurses working at hospitals in West Shoa, Ethiopia, 2022.

Variables	Category	Frequency (*n* = 253)	Percentage
Lack of teaching materials to use preoperatively can affect the delivery of preoperative patients teaching.	Agree	142	56.1
	Disagree	111	43.9
Lack of training on preoperative teaching is a factor to proper delivery of preoperative teaching to patients before undergoing surgery.	Agree	133	52.6
	Disagree	120	47.4
A preoperative patient teaching is affected by the daily workload of the nurse.	Agree	131	51.8
	Disagree	122	48.2
Lack of available time among nursing staff can affect the delivery of preoperative teaching to patients undergoing surgery.	Agree	140	55.3
	Disagree	113	44.7
The shortage of nursing staff is a factor affecting the practice of preoperative patient teaching.	Agree	142	56.1
	Disagree	111	43.9
Language can be a barrier in delivering preoperative patient teaching.	Agree	136	53.8
	Disagree	117	46.2
The preoperative patients’ teaching can be affected by the tight operations scheduled daily in the operating theater.	Agree	131	51.8
	Disagree	123	48.2
Preoperative patient teaching is a challenge among critically ill patients.	Agree	132	52.8
	Disagree	121	48.2
Patient and family anxiety about the outcome of surgery	Agree	129	51.0
	Disagree	124	49.0
Feeling shy to ask nurses certain question is a factor to the practice of preoperative patients teaching.	Agree	131	51.8
	Disagree	122	48.2
Complexity of patients’ status is a factor to the practice of preoperative patients teaching.	Agree	148	58.5
	Disagree	105	41.5

### Factors associated with preoperative patient teaching practice

To assess the factors associated with nurses’ preoperative patient teaching practice, a bivariate analysis was performed. Accordingly, marital status, educational level, work experience, lack of teaching materials, lack of training, workload, time constraints, insufficient staffing, language barrier, tight operation schedule, severity of patient cases, anxiety, feeling too shy to ask questions, and complexity of patient status were significantly associated with nurses preoperative teaching practice (0.25). Variables associated with the dependent variable (*p*-value <0.25) were selected and subjected to multivariate logistic regressions analysis (Tables 3, 4).

**Table 3:** Bivariable logistic regression result of factors associated with preoperative teaching practice among nurses working at West Shoa, Ethiopia, 2022.

Variables	Category	Practice status		COR with 95% CI	*p*-value
		Poor	Good		
Age	18–35	139 (60.2%)	92 (39.8%)	1.04 (0.43–2.5)	0.921
	≥35	13 (59.1%)	9 (40.9%)	1	
Sex	Male	77 (58.3%)	55 (41.7%)	0.85 (0.51–1.42)	0.554
	Female	75 (62.0%)	46 (38.0%)	1	
Marital status	Single	42 (47.7%)	46 (52.3%)	1.06 (0.33–3.4)	0.916
	Married	104 (68.4%)	48 (31.6%)	2.5 (0.8–7.9)	0.112
	Other*	6 (46.2%)	7 (53.8%)	1	
Religion	Orthodox	37 (50.7%)	36 (49.3%)	0.6 (0.2–1.8)	0.394
	Protestant	105 (64.0%)	59 (36.0%)	1.06 (0.37–0.3)	0.904
	Other**	10 (62.5%)	6 (37.5%)	1	
Educational level in nursing	Diploma	17 (73.9%)	6 (26.1%)	3.46 (0.96–12.4)	0.057
	BSc	126 (60%)	84 (40%)	1.83 (0.73–4.6)	0.98
	MSc	9 (45%)	11 (55%)	1	
Work experience in year	≤9	103 (56.9%)	78 (43.1%)	0.62 (0.35–1.10)	0.104
	≥10	49 (68.1%)	23 (31.9%)	1	
Lack of teaching materials	Agree	105 (73.9%)	37 (26.1%)	3.86 (2.27–6.57)	0.001
	Disagree	47 (42.3%)	64 (57.7%)	1	
Lack of training	Agree	88 (73.3%)	32 (26.7%)	2.96 (1.75–5.0)	0.001
	Disagree	64 (48.1%)	69 (51.9%)	1	
Workload	Agree	109 (77.9%)	31 (22.1%)	5.7 (3.3–9.9)	0.001
	Disagree	43 (38.1%)	70 (61.9%)	1	
Shortage of time	Agree	99 (70.7%)	41 (29.3%)	2.7 (1.6–4.6)	0.001
	Disagree	53 (46.9%)	60 (53.1%)	1	
Insufficient staffing	Agree	101 (71.1%)	41 (28.9%)	2.89 (1.7–4.88)	0.001
	Disagree	51 (45.9%)	60 (54.1%)	1	
Language barrier	Agree	100 (73.5%)	36 (36.5%)	3.47 (2.05–5.89)	0.001
	Disagree	52 (44.4%)	65 (55.6%)	1	
Tight operation schedule	Agree	95 (74.5%)	36 (25.5%)	3.0 (1.8–5.1)	0.001
	Disagree	57 (46.7%)	65 (53.3%)	1	
Severity of patient cases	Agree	99 (75.0%)	33 (25.0%)	3.85 (2.26–6.56)	0.001
	Disagree	53 (43.8%)	68 (56.2%)	1	
Anxiety	Agree	98 (76%)	31 (24%)	4.098 (2.39–7.01)	0.001
	Disagree	54 (43.5%)	70 (56.5%)	1	
Shy to ask question	Agree	92 (70.2%)	39 (29.8%)	2.43 (1.45–4.08)	0.001
	Disagree	60 (49.2%)	62 (50.8%)	1	
Complexity of patient status	Agree	113 (76.4)	35 (23.6)	5.46 (3.15–9.45)	0.001
	Disagree	39 (37.1%)	66 (62.9%)	1	

Other** = Muslim and Catholic, other* = widowed and divorced.

**Table 4:** Bivariable and multivariable logistic regression result of factors associated with preoperative teaching practice among nurses working at West Shoa, Ethiopia, 2022.

Variables	Category	Practice status		COR with 95% CI	AOR with 95% CI
		Poor	Good		
Marital status	Single	42 (47.7%)	46 (52.3%)	1.06 (0.33–3.4)	1.2 (0.31–5.2)
	Married	104 (68.4%)	48 (31.6%)	2.5 (0.8–7.9)	0.95 (0.25–3.5)
	Other*	6 (46.2%)	7 (53.8%)	1	1
Educational level in nursing	Diploma	17 (73.9%)	6 (26.1%)	3.46 (0.96–12.4)	4.68 (0.79–27.7)
	BSc	126 (60%)	84 (40%)	1.83 (0.73–4.6)	1.18 (0.3–4.6)
	MSc	9 (45%)	11 (55%)	1	1
Experience in year	≤9	103 (56.9%)	78 (43.1%)	0.62 (0.35–1.10)	0.82 (0.33–2.0)
	≥10	49 (68.1%)	23 (31.9%)	1	1
Lack of teaching materials	Agree	105 (73.9%)	37 (26.1%)	3.86 (2.27–6.57)	3.29 (1.57–6.92)*
	Disagree	47 (42.3%)	64 (57.7%)	1	1
Lack of training	Agree	88 (73.3%)	32 (26.7%)	2.96 (1.75–5.0)	2.87 (1.65–4.97)**
	Disagree	64 (48.1%)	69 (51.9%)	1	1
Workload	Agree	109 (77.9%)	31 (22.1%)	5.7 (3.3–9.9)	8.60 (3.91–19.5)**
	Disagree	43 (38.1%)	70 (61.9%)	1	1
Shortage of time	Agree	99 (70.7%)	41 (29.3%)	2.7 (1.6–4.6)	2.48 (1.43–4.27)**
	Disagree	53 (46.9%)	60 (53.1%)	1	1
Insufficient staffing	Agree	101 (71.1%)	41 (28.9%)	2.89 (1.7–4.88)	2.36 (1.07–5.21)*
	Disagree	51 (45.9%)	60 (54.1%)	1	1
Language barrier	Agree	100 (73.5%)	36 (36.5%)	3.47 (2.05–5-89)	3.16 (1.47–6.78)*
	Disagree	52 (44.4%)	65 (55.6%)	1	1
Tight operation schedule	Agree	95 (74.5%)	36 (25.5%)	3.0 (1.8–5.1)	1.63 (0.72–3.68)
	Disagree	57 (46.7%)	65 (53.3%)	1	1
Severity of patient cases	Agree	99 (75.0%)	33 (25.0%)	3.85 (2.26–6.56)	5.88 (5.88–12.7)**
	Disagree	53 (43.8%)	68 (56.2%)	1	1
Anxiety	Agree	98 (76%)	31 (24%)	4.098 (2.39–7.01)	4.18 (1.95–8.92)**
	Disagree	54 (43.5%)	70 (56.5%)	1	1
Shy to ask question	Agree	92 (70.2%)	39 (29.8%)	2.43 (1.45–4.08)	1
	Disagree	60 (49.2%)	62 (50.8%)	1	1.85 (0.85–4.04)
Complexity of patient status	Agree	113 (76.4)	35 (23.6)	5.46 (3.158–9.45)	4.46 (2.09–9.53)**
	Disagree	39 (37.1%)	66 (62.9%)	1	1

*p-value < 0.05, **p-value < 0.001, 1-reference category, other* = widowed and divorced.

After adjusting for nine variables, the lack of teaching materials, lack of training, workload, time constraints, shortage of nurses, language barrier, severity of patient cases, anxiety, and complexity of patients’ status were significantly associated with nurses’ preoperative teaching practice (Table 5).

**Table 5:** Preoperative patient teaching practice among nurses working at hospitals in West Shoa Zone, Ethiopia, 2022.

Variables	Never	Rarely	Some times	Always
I teach patient about routine surgical procedures before surgery	0	20 (7.9%)	71 (28.1%)	162 (64%)
I teach patient’s family members about perioperative environment before surgery.	8 (3.2%)	52 (20.6%)	37 (14.6%)	156 (61.7%)
I teach patients undergoing surgery within a specific time before surgery.	15 (5.9%)	51 (20.2%)	34 (14.4%)	153 (60.5%)
I teach patients about the fasting time before undergoing surgery.	13 (5.1%)	45 (17.8%)	36 (14.2%)	159 (62.8%)
I teach the patients about the postoperative nutritional requirement	6 (2.4%)	45 (17.8%)	36 (14.2%)	166 (65.6%)
I teach the patients about Preoperative medication and feelings after receiving the drug.	10 (4.0%)	46 (18.2%)	43 (17.0%)	154 (60.9%)
I teach the patient about postoperative expectations.	9 (3.6%)	60 (23.7%)	27 (10.7%)	157 (62.1%)
I provide explanations regarding the types of anesthesia to use during surgery.	12 (4.7%)	54 (21.3%)	34 (13.5%)	153 (60.5%)
I provide the teaching regarding emptying of the bladder before going to the theater	9 (3.6%)	49 (19.4%)	38 (15.0%)	157 (62.0%)
I provide the teaching regarding details about catheterization	17 (6.7%)	48 (19.0%)	30 (11.9%)	158 (62.5%)
I provide teaching regarding shaving and its importance	10 (4.0%)	58 (22.9%)	27 (10.7%)	158 (62.4%)
I provide teaching regarding the perioperative environment to patients before surgery.	14 (5.5%)	54 (21.3%)	35 (13.8%)	150 (59.4%)
I explain to the patient before undergoing surgery about the management of postoperative pain.	21 (8.3%)	46 (18.2%)	34 (13.4%)	152 (60.1%)
I provide teaching to patients undergoing surgery about skin hygiene on the day of surgery.	15 (5.9%)	33 (13.4%)	65 (25.7%)	140 (55.0%)
I teach patients undergoing surgery that they should leave valuables and remove all jewelry before going into the operating room for surgery.	22 (8.7%)	57 (22.5%)	37 (14.6%)	137 (54.2%)
I use different methods when I provide preoperative teaching to patients before surgery.	14 (5.5%)	67 (26.5%)	44 (17.4%)	128 (50.6%)
I use teaching materials when providing preoperative patient teaching.	25 (9.9%)	59 (23.3%)	38 (15.0%)	131 (51.8%)
I teach the patients undergoing surgery about postoperative deep breathing and coughing.	12 (4.7%)	62 (24.5%)	42 (16.6%)	137 (54.2%)
I teach the patients undergoing surgery about the time for postoperative early mobilization.	10 (4.0%)	55 (21.7%)	36 (14.2%)	152 (60.1%)

## Discussion

In this study, we attempted to identify preoperative patient teaching practice and associated factors among nurses working at hospitals in West Shoa Zone. According to this study, 60.1% of study participants demonstrated poor preoperative patient teaching practices. Factors such as lack of teaching materials, lack of training, workload, time constraints, insufficient staffing, language barrier, severity of patient cases, patient and family’s anxiety, and complexity of patients’ status were statistically associated with preoperative patient teaching practice.

This finding is in line with studies conducted in East Amhara and Northwest Amhara Comprehensive Specialized Referral Hospitals, that is, only 61.5 and 53.7% of nurses, respectively, were found to demonstrate poor practices in delivering preoperative patient education. The similarities might be due to the study setting, the study population, sociodemographic traits, and curriculum.

This result is again supported by the studies conducted in Nigeria which revealed a positive attitude to patients’ preoperative education; however, the practice was not effective (33). Despite differences in socioeconomic status and level of health sector development, the current study and the Nigerian study may be similar due to the use of a similar study population (staff nurse) and study design. This is also in agreement with a study conducted in Gamo and Gofa, southern Ethiopia which revealed that only 36.6% of patients received good quality preoperative education (34).

However, this finding is better than that of study conducted in China which revealed that 46.5% of nurses did not provide all necessary preoperative information to patients (10). This inconsistency might be due to socioeconomic differences between the two countries. The results of our study are better than those of a study conducted in Cyprus which revealed that approximately 88.8% of nurses offered preoperative patient education and only 11.2% offered none (12). This discrepancy might be due to the nurse-to-patient ratio, training, and use of different data collection tool. Study conducted in Iran also revealed worse results than ours: 84.9% of nurses always educate their patients, and 15.1% do not always educate their patients (28). The socioeconomic differences between those two countries may be the cause of this discrepancy.

On the other hand, this result is lower than that of study conducted in Rwanda, which found that 97.3% of nurses demonstrate poor preoperative patient teaching practices (14). This discrepancy might be due to study setting, sample size, or educational level of nurses since most Rwandan nurses were diploma holders rather than BSc holders as was the case in our current study.

The findings of the current study revealed that lack of teaching materials was significantly associated with nurses’ preoperative teaching practices. Respondents who agreed that lack of teaching material affect preoperative patient teaching practice were three times more likely to demonstrate poor practice than their counterparts. This study is comparable with a study conducted in China and Rwanda which revealed that limited teaching resources affected preoperative teaching provided to surgical patients (10, 14). A possible explanation could be that the scarcity of teaching materials in both developed and developing nations has a negative impact on delivery of preoperative education.

Study showed that lack of training was significantly associated with nurses’ preoperative teaching practice and respondents who agreed this was a factor practicing preoperative teaching were three times more likely to demonstrate poor practice compared to those who disagreed. This result is supported by study conducted in China and Nigeria which showed that a lack of training is among the top factors affecting preoperative education provided for surgical patients (10, 27). This can be related to the increased knowledge of operation details and specific perioperative care gained from attending relevant surgical training courses; as a result, they were much more eager and satisfied to use their newly acquired knowledge to teach and provide more preoperative information to patients. This suggests that the responsible bodies are prepared to deliver service and in-service training programs for nurses on preoperative patient teaching practice based on the most recent evidence-based global and national recommendations. Similarly, work load was among the variables significantly associated with nurses’ preoperative teaching practice and those who agreed that workload is a factor in practicing preoperative teaching were 8.6 times more likely to demonstrate poor practice compared to their counterparts. This finding is in agreement with a studies conducted in Malaysia, China, Israel, Southern Alberta, Houston, Nigeria, and Rwanda (10, 14, 35–38). The argument that could be made is that in both developed and developing nations, workload prevents nurses from delivering preoperative patient education.

Insufficient staffing was another factor significantly associated with preoperative patient teaching practice. Study participants who agreed that insufficient staffing was a factor affecting preoperative patient teaching practice were twice likely to demonstrate poor preoperative patient teaching practice compared to the opposite one. This finding is supported by a studies conducted in Canada, Nigeria, and Rwanda (14, 27, 39). The argument that could be made is that both in developed and in developing nations, the shortage of nurses is a problem in delivering preoperative education for patients.

This study found that those nurses who had enough time were significantly associated with good preoperative patient teaching practice as compared with those nurses who did not have enough time. This study was consistent with the studies done in Hong Kong, Saudi Arabia, and northern Ethiopia (10, 20, 40). The possible explanation could be a lack of time due to the high burden of surgical cases following at the current time, as nurses’ engagement in preoperative education and time constraints in clinical practice prevented nurses from performing any patient education activities. This possible explanation is supported by the World Bank report, “The Health Workforce in Ethiopia,” which shows Ethiopia is one of the countries with a very low health workforce, five times below the minimum threshold of 4.45 per 1,000 population set by the World Health Organization to achieve the SDG Health.

Language barriers are another factor significantly associated with preoperative teaching practice and respondents who agreed that the language barrier was a factor were three times more likely to demonstrate poor practice than their counter parts. This finding is supported by studies conducted in China and Nigeria which showed that language barrier was among the highest ranked factors affecting the preoperative teaching practice. A possible justification might be that language is a powerful tool for teaching and learning in all contexts because it facilitates the transference of ideas. The severity of cases was also significant factor associated with quality of teaching practice.

Study participants who agreed it was difficult to teach critically ill patients were six times more likely to demonstrate poor practice than those who disagreed. This finding was also supported by study conducted in Cameroon which revealed that severe medical condition of patients was one of the major factors affecting preoperative teaching practice (25). This may be because teaching patients with serious medical conditions is challenging, and they may even be in comas.

Anxiety among patients and their families was another factor significantly associated with preoperative teaching practice. Respondents who agreed that the patients and their families’ anxiety is a barrier to the practice of preoperative teaching were four times more likely to demonstrate poor practice compared to their counterparts. This result is supported by a study conducted in Rwanda which revealed that 54.1% of respondents agreed that patient and family anxiety about the outcomes of surgery is a barrier to the practice of preoperative patient teaching (14). A possible explanation could be that they are afraid of the outcomes of surgery and are unable to follow or understand what the nurses are teaching them.

The complexity of patients’ status is significantly associated with preoperative teaching practice. The result of this study showed that study participants who agreed that complexity of patients’ status affected preoperative patient teaching practice were four times more likely to demonstrate than their counterparts. This finding is supported by a study conducted in Houston which revealed that the complexity of patients’ status was major factors influencing patient education (39). A possible reason for this finding might be that patients with more than one medical condition find it difficult to concentrate on what nurses are telling them regarding one surgical procedure because they are thinking about another of their conditions at the same time.

### Strength and limitation

The study included different levels of hospitals from primary hospital to tertiary hospitals which increase the study’s representativeness. It was unique in study area as it comprehensively examined factors affecting preoperative teaching practice by examining many variables from different dimensions. As a limitation, the study used only a cross-sectional quantitative study design, which may not be sufficient to find all possible factors associated with preoperative patient teaching practice among nurses. In addition, the cause-and-effect relationship cannot be confirmed in this study because the research design was cross-sectional. Another limitation of the study is due to the resource constraint; we could not conduct an observational data collection method.

## Conclusion and recommendations

The good preoperative patient teaching practices demonstrated by nurses working in hospitals in the West Shoa Zone were found to be inadequate. Preoperative education practices was significantly associated with lack of teaching materials, a lack of training, a heavy workload, time constraints, insufficient staffing, a language barrier, the severity of patient cases, anxiety about postoperative outcomes, and the complexity of patients’ status. Establish preoperative patient teaching programs for nurses that exchange experiences. It is better to strengthen training, adequate staffing, and equip wards with standardized guidelines and teaching materials and motivate and create a safe working environment to provide adequate preoperative teaching.

## Data Availability

The raw data supporting the conclusions of this article will be made available by the authors, without undue reservation.
